# The 1-Week and 8-Month Effects of a Ketogenic Diet or Ketone Salt Supplementation on Multi-Organ Markers of Oxidative Stress and Mitochondrial Function in Rats

**DOI:** 10.3390/nu9091019

**Published:** 2017-09-15

**Authors:** Wesley C. Kephart, Petey W. Mumford, Xuansong Mao, Matthew A. Romero, Hayden W. Hyatt, Yufeng Zhang, Christopher B. Mobley, John C. Quindry, Kaelin C. Young, Darren T. Beck, Jeffrey S. Martin, Danielle J. McCullough, Dominic P. D’Agostino, Ryan P. Lowery, Jacob M. Wilson, Andreas N. Kavazis, Michael D. Roberts

**Affiliations:** 1 School of Kinesiology, Auburn University, Auburn, AL 36849, USA; wck0007@auburn.edu (W.C.K.); pwm0009@auburn.edu (P.W.M.); xzm0012@auburn.edu (X.M.); mzr0049@auburn.edu (M.A.R.); hwh0001@auburn.edu (H.W.H.); moblecb@auburn.edu (C.B.M.); kyoung@auburn.vcom.edu (K.C.Y.); dbeck@auburn.vcom.edu (D.T.B.); jmartin@auburn.vcom.edu (J.S.M.); dmccullough@auburn.vcom.edu (D.J.M.); 2 Department of Biological Sciences, Auburn University, Auburn, AL 36849, USA; yzz0095@auburn.edu; 3 Department of Human Health Performance, University of Montana, Missoula, MT 59812, USA; john.quindry@mso.umt.edu; 4 Department of Cell Biology and Physiology, Edward via College of Osteopathic Medicine—Auburn Campus, Auburn, AL 36849, USA; 5 Department of Molecular Pharmacology and Physiology, University of South Florida, Tampa, FL 33620, USA; dagostino.dominic1@gmail.com; 6 Applied Sports Performance Institute, Tampa, FL 33607, USA; rlowery@theaspi.com (R.P.L.); jwilson@theaspi.com (J.M.W.)

**Keywords:** ketogenic dieting, ketone salts, skeletal muscle, brain, liver, oxidative stress, mitochondria

## Abstract

We determined the short- and long-term effects of a ketogenic diet (KD) or ketone salt (KS) supplementation on multi-organ oxidative stress and mitochondrial markers. For short-term feedings, 4 month-old male rats were provided isocaloric amounts of KD (*n* = 10), standard chow (SC) (*n* = 10) or SC + KS (~1.2 g/day, *n* = 10). For long-term feedings, 4 month-old male rats were provided KD (*n* = 8), SC (*n* = 7) or SC + KS (*n* = 7) for 8 months and rotarod tested every 2 months. Blood, brain (whole cortex), liver and gastrocnemius muscle were harvested from all rats for biochemical analyses. Additionally, mitochondria from the brain, muscle and liver tissue of long-term-fed rats were analyzed for mitochondrial quantity (maximal citrate synthase activity), quality (state *3* and *4* respiration) and reactive oxygen species (ROS) assays. Liver antioxidant capacity trended higher in short-term KD- and SC + KS-fed versus SC-fed rats, and short-term KD-fed rats exhibited significantly greater serum ketones compared to SC + KS-fed rats indicating that the diet (not KS supplementation) induced ketonemia. In long term-fed rats: (a) serum ketones were significantly greater in KD- versus SC- and SC + KS-fed rats; (b) liver antioxidant capacity and glutathione peroxidase protein was significantly greater in KD- versus SC-fed rats, respectively, while liver protein carbonyls were lowest in KD-fed rats; and (c) gastrocnemius mitochondrial ROS production was significantly greater in KD-fed rats versus other groups, and this paralleled lower mitochondrial glutathione levels. Additionally, the gastrocnemius pyruvate-malate mitochondrial respiratory control ratio was significantly impaired in long-term KD-fed rats, and gastrocnemius mitochondrial quantity was lowest in these animals. Rotarod performance was greatest in KD-fed rats versus all other groups at 2, 4 and 8 months, although there was a significant age-related decline in performance existed in KD-fed rats which was not evident in the other two groups. In conclusion, short- and long-term KD improves select markers of liver oxidative stress compared to SC feeding, although long-term KD feeding may negatively affect skeletal muscle mitochondrial physiology.

## 1. Introduction

Ketogenic diets (KD) are high fat, moderate protein and low carbohydrate diets that have been associated with a myriad of health benefits including weight loss/management, neurological improvements (e.g., treatment of epilepsy and certain brain cancers) and longevity [1,2]. Rodent models have also indicated that ketogenic dieting increases lifespan [3,4], although the specific mechanisms underpinning these observations have not been well elucidated. Excessive tissue accretion of oxidative stress due to repetitive insults from reactive oxygen species (ROS) are a hallmark signature of the aging process [5,6]. Mitochondria bear the brunt of oxidative damage in the lesions to mitochondrial DNA and are reported to be as much as 10-fold higher than in nuclear DNA [7]. Hence, dietary manipulations that mitigate mitochondrial oxidative stress may serve to mitigate the aging process and optimize cellular function. 

Interestingly, oxidative stress defense and improved mitochondrial quantity/quality are potential mechanisms through which KD feeding may confer physiological benefits [8,9]. For instance, hippocampal mitochondrial biogenesis in rodents has been reported after four weeks of KD feeding [10]; this is a phenomenon which could occur via KD-induced AMPK pathway signaling [11,12]. KD feeding has also been shown to decrease ROS and hydrogen peroxide (H_2_O_2_) production in rodent tissues by increasing uncoupling protein-mediated proton conductance and/or increasing glutathione biosynthesis [10,13,14,15]. With regard to the later mechanism, Milder and Patel [8] have theorized that KD-induced increases in ROS defense is related to an acute stimulation of 4-hydroxy-2-nonenal (4-HNE) or H_2_O_2_ which, in turn, stimulates the nuclear factor (erythroid-derived 2)-like 2 (Nrf2) transcription factor to translocate into the nucleus. Nrf2 has specifically been shown to upregulate the mRNA expression glutamate-cysteine ligase (GCL) subunits which is the rate limiting enzyme in glutathione biosynthesis [16,17]. In support of this model, Milder and colleagues [18] reported that KD feeding stimulates H_2_O_2_ and 4-HNE production in the hippocampus of rodents within a 1–3 days period, and this leads to Nrf2 nuclear translocation within 7 days as well as subsequent increases in the expression of ROS-protective genes that promote oxidative stress resistance during 1–3 weeks of feeding. While it is currently unclear whether these redox adaptations are due to a direct effect of the diet (i.e., dietary ketosis) or an indirect effect of the diet (i.e., weight loss, lower insulin levels, etc.), it is notable that several beneficial physiological effects of KD feeding are independent of weight loss. In this regard, potential antioxidant effects of ketogenic dieting may be stimulated by the consequent metabolites of ketone bodies, beta-hydroxybutyrate (BHB) and acetoacetate (AcAc). For instance, ROS production is decreased and resistance to a H_2_O_2_-induced stress is conferred when ketones are introduced in cell culture models [19,20]. Thus, it is also possible that exogenous ketone supplementation may also have a direct effect in terms of attenuating ROS production and quenching free radical chain reactions.

Based upon the aforementioned supporting literature, it stands to reason that KD feeding or dietary ketone salt supplementation may mitigate ROS production or attenuate oxidative stress over long-term periods which, in turn, may improve mitochondrial quality. Therefore, the purpose of this investigation was to examine if short-term (i.e., 1 week) and/or long-term (i.e., 8 months) KD feeding or BHB salt supplementation affect skeletal muscle, brain and/or liver measures of oxidative stress and mitochondrial function. Our a priori hypothesis was that KD feeding and ketone salt supplementation would acutely and chronically enhance the expression of endogenous antioxidants and, in long-term rats, this would translate to a reduction in tissue oxidative stress markers and enhanced mitochondrial quantity and quality.

## 2. Materials and Methods

### 2.1. Rats in 1 Week Experiment

All experimental procedures were approved by Auburn University’s Institutional Animal Care and Use Committee (IACUC, protocol # 2016-2814, approval date 6 January 2016). Male Fisher 344 rats at 4 months of age (~360 g) were purchased (Harlan Laboratories, Indianapolis, IN, USA) and allowed to acclimate in the animal housing facility at Auburn University for 1 week prior to experimentation. During acclimation, rats were provided standard rodent chow (SC; 24% (% kcal) protein, 58% CHO (2.8% fiber *w*/*w*), 18% fat; Teklad Global #2018 Diet, Harlan Laboratories) and water ad libitum in a maintained ambient temperature and constant 12 h light: 12 h dark cycle. For a 1 week period following acclimation, rats were provided isocaloric-isonitrogenous-isofibrous amounts of one of three diets:

(1) 10 rats (SC) were provided with 20 g/day of the aforementioned SC given during the acclimation phase.
(2) 10 rats (KD) were provided with 16 g/day of a commercially designed KD (Harlan Tekland diet #10787; Harlan Laboratories, Indianapolis, IN, USA) that was designed to induce nutritional ketosis and has been used previously by our laboratory [21]. Casein protein (Optimum Nutrition Inc., Downers Grove, IL, USA) and cellulose powder (Allergy Research Group, Alameda, CA, USA) were added to better compensate for between group differences in protein and fiber content (added protein and fiber were 23.5% of the modified diet). The diet specifications (following modifications) were as follows: 4.15 kcal/g, 23% protein, 10% carbohydrate (2.9% fiber *w*/*w*), and 67% fat. Medium chain triglycerides, flaxseed oil and canola oil were prominent fat sources in the parent KD.
(3) 10 rats (SC + KS) were provided with 20 g/day of the aforementioned SC, along with sodium BHB salt (DL-3 sodium hydroxybutyric acid, 5.8 kcal/g; NNB Nutrition, Lewisville, TX, USA) which were added to drinking water for ad libitum consumption with the intent to deliver ~2.2 g/day. This dosing schedule was designed with the intent of delivering a human-equivalent dose of 40 g/day dose as a “loading phase” per the body surface area rat-to-human conversions of Reagan-Shaw et al. [22] assuming the average rat weight of 400 g and the average human weight of 80 kg.

Notably, body masses were measured every other day, and food weights were measured daily.

### 2.2. Rats in 8 Months Experiment

As with the procedures outlined above, 8 months experimental procedures were approved by Auburn University’s IACUC (protocol # 2016-2814, approval date 6 January 2016). Male Fisher 344 rats 4 months of age (~360 g) were purchased (Harlan Laboratories, Indianapolis, IN, USA) and allowed to acclimate in the animal housing facility for 1 week prior to experimentation in the same manner as described above. For an 8 months period following acclimation, rats were provided with isocaloric-isonitrogenous-isofibrous amounts of the three diets described above (SC *n* = 8, KD *n* = 8, SC + KS *n* = 8). In the SC + KS group, KS were added to water bottles attempting to deliver ~2.2 g/day for the first week as a “loading phase”, then 0.3 g/day as a “maintenance phase” for the remaining duration. This dosing schedule was designed with the intent of delivering a human-equivalent dose of 40 g/day dose during the loading phase and a 10 g/day dose during the maintenance phase per the body surface area rat-to-human conversions of Reagan-Shaw et al. described above. One SC + KS rat inexplicably lost >20% body mass during the first two months of treatment and, thus, was euthanized for humane reasons and not included in the analyses. One SC rat experienced rapid weight loss towards the end of the 8 months intervention. This rat presented large intra-abdominal tumors upon necropsy and was also not included in the analysis. Thus, final n-sizes were SC *n* = 7, KD *n* = 8 and SC + KS *n* = 7. Notably, bodyweights were recorded weekly, food intakes were recorded daily, and water was measured daily in order to ascertain the amount of KS ingested by the SC + KS group.

### 2.3. Rotarod Performance in 8 Month-Fed Rats

Rotarod performance is used in rodent studies to assess a combination of balance, grip strength, motor coordination and muscular endurance [23]. In 8 month-fed rats, rotarod performance was assessed at 2, 4, 6 and 8 months into the intervention using a single-lane device (Product#: ENV-571R; Med Associates Inc., Saint Albans City, VT, USA). Briefly, all assessments took place during the beginning of the rat light cycle (i.e., 0600–0800) whereby rats were placed on the device and the motorized rotor was initiated at a progressive speed from 4.0 to 40.0 r/min. An automated timer tracked time spent on the rod and, once the rats fatigued and dismounted from the rod, a laser beam break stopped the timer. 

### 2.4. Necropsies and Tissue Preparation in Rats from Both Feeding Experiments

On the morning of necropsies (0500–0600), rats were food-deprived but provided water ad libitum. Rats were then transported from the campus vivarium to the School of Kinesiology and allowed to acclimate for 2–3 h. Thereafter, rats were euthanized under CO_2_ gas in a 2 L induction chamber (VetEquip, Pleasanton, CA, USA). Following euthanasia, a final body mass was recorded, and blood was collected from the heart using a 22 gauge syringe. Collected blood was placed in a 6 mL serum separator tube and allowed to clot, centrifuged at 3500 g for 10 min, and resultant serum was aliquoted into 2.7 mL microcentrifuge tubes for storage at −80 °C until analysis. The gastrocnemius, brain, and liver were dissected out. In 1 week-fed rats, approximately 50 mg from each tissue was immediately placed in RNA/DNA Shield (Zymo Research, Irvine, CA, USA) and stored at 4 °C until RNA isolation using Trizol-based methods (described in Section 2.5), whereas the remainder of the tissue was flash frozen in liquid nitrogen and stored at −80 °C until total antioxidant analysis. In 8 month-fed rats, necropsies were carried out exactly as detailed above with the exception being that approximately 800 mg from the right gastrocnemius muscle, 1000 mg of liver and 500 mg of whole-brain (without cerebellum) was immediately used for mitochondria isolation as described below. The remainder of the tissue was flash frozen in liquid nitrogen and stored at −80 °C until Western blotting, GSH/GSSG and total antioxidant capacity analyses described below. 

### 2.5. RNA Isolation, cDNA Synthesis and Real-Time Polymerase Chain Reaction (RT-PCR) for 1 Week-Fed Rat Tissues

Muscle/brain/liver stored in RNA/DNA Shield described above were placed in 10 volumes (500 µL) Ribozol (Ameresco, Solon, OH, USA) in a 2.7 mL microcentrifuge tube and were homogenized with a tight-fitting pestle. Phase separation (for RNA isolation) was achieved according to manufacturer’s instructions. Following RNA precipitation and pelleting, pellets were resuspended in 30 µL of RNase-free water, and RNA concentrations were determined in duplicate at an absorbance of 260 nm by using a NanoDrop Lite (Thermo Scientific, Waltham, MA, USA). For cDNA synthesis, 1 µg of muscle/liver/brain RNA was reverse transcribed into cDNA for RT-PCR analyses with a commercial qScript cDNA SuperMix (Quanta Biosciences, Gaithersburg, MD, USA). RT-PCR was performed with gene-specific primers and SYBR green-based methods in a RT-PCR thermal cycler (Bio-Rad, Hercules, CA, USA). Primers were designed with open-sourced software (Primer3Plus, Cambridge, MA, USA), and melt curve analyses demonstrated that one PCR product was amplified per reaction. The forward and reverse primer sequences are as follows: Glutamate-Cysteine Ligase Modifier Subunit (Gclm): forward primer 5′-ACATTGAAGCCCAGGAGTGG-3′, reverse primer 5′-CGATGACCGAGTACCTCAGC-3′; Glutamate-Cysteine Ligase Catalytic Subunit (Gclc): forward primer 5′-GAGATGCCGTCTTACAGGGG-3′, reverse primer 5′-TTGCTACACCCATCCACCAC-3′. Catalase (Cat): forward primer 5′-TTAACGCGCAGATCATGCA-3′, reverse primer 5′-CAAGTTTTTGATGCCCTGGT-3′. Glutathione peroxidase (Gpx): forward primer 5′-TCTGCACACTCCCAGACAAG-3′, reverse primer 5′-AGTCACCCATCACCGTCTTC-3′. Superoxide dismutase 2 (Sod2): forward primer 5′-TTAACGCGCAGATCATGCA-3′, reverse primer 5′-CCTCGGTGACGTTCAGATTGT-3′. Fold-change values from SC rats were performed using the 2^−ΔΔCT^ method where 2^ΔCT^ = (housekeeping gene (HKG) CT − gene of interest CT) and 2^−ΔΔCT^ (or fold-change) = (2^ΔCT^ value for each rat/2^ΔCT^ group average of SC). Of note, 18S ribosomal rRNA (18S) was used as a HKG given that it remained stable across all treatments (primer sequence: forward primer 5′-AAACGGCTACCACATCCAAG-3′, reverse primer 5′-CCTCCAATGGATCCTCGTTA-3′).

### 2.6. Tissue Total Antioxidant and Serum BHB Assays for All Rats

Commercial colorimetric assay kits were used to determine muscle/liver/brain total antioxidant capacity (Antioxidant assay kit #709001; Cayman Chemical, Ann Arbor, MI, USA) and serum BHB levels (BHB colorimetric assay kit #700190; Cayman Chemical), respectively, according to manufacturer’s instructions. For total antioxidant capacity determination, approximately 100 mg of frozen muscle/brain/liver was homogenized in kit assay buffer and centrifuged according to manufacturer’s instructions, and supernatants were assayed. Given that brain tissue in 8 month-fed rats was devoted to mitochondrial assays and Western blotting, brain total antioxidant capacity determination was not performed because of lack of available tissue. Following assay execution, all plates were read in a UV-Vis microplate reader (BioTek Synergy H1 Multi-Mode Reader; BioTek, Winooski, VT, USA) at absorbance readings according to manufacturer’s recommendations.

### 2.7. Western Blot Analysis in 8 Month-Fed Rat Tissues

Muscle/brain/liver was removed from −80 °C storage and crushed on a liquid nitrogen-cooled stage. Approximately 50 mg of tissue from each tissue sample was placed in 500 μL of 1× non-denaturing cell lysis buffer (Cell Signaling, Danvers, MA, USA) with added protease inhibitors (1 μg/mL leupeptin) and phosphatase inhibitors (2.5 mM sodium pyrophosphate, 1 mM β-glycerophoshate, 1 mM sodium orthovanadate) and was homogenized in microcentrifuge tubes using tight-fitting pestles. Samples were centrifuged at 500 *g* for 5 min at 4 °C. Supernatants were then subjected to a protein assay using a commercial bicinchoninic acid assay (Thermo Scientific) and were prepared for Western blotting using 4× Laemmli reducing buffer at 2 μg/μL. Subsequently, 20 μL of prepped samples were loaded onto precast 12% SDS-polyacrylamide gels (Bio-Rad) and were subjected to electrophoresis (200 V at 75 min) using premade 1× SDS-PAGE running buffer (CBS Scientific, San Diego, CA, USA). Proteins were transferred to polyvinylidene difluoride membranes (Bio-Rad), and membranes were stained with Ponceau S following transfers to ensure even loading and transfer between samples. Membranes were then blocked with 5% nonfat milk powder diluted in TBS with 0.1% Tween-20 (TBST) for 1 h at room temperature. Primary antibodies directed against the proteins of interest were incubated with membranes overnight at 4 °C in TBST with 5% BSA added. Primary antibodies were used to detect whole-tissue 4-HNE (Abcam, Cambridge, MA, USA), superoxide dismutase 2 (SOD2; GeneTex, Irvine, CA, USA), Catalase (Cat; GeneTex), glutathione peroxidase (Gpx; GeneTex) and protein carbonyls (Oxyblot kit; EMD Millipore; Bellirica, MA, USA). On the following day, membranes were incubated with anti-rabbit or anti-mouse IgG secondary antibodies diluted in TBST with 5% BSA added (1:2000; Cell Signaling) at room temperature for 1 h prior to membrane development. Membrane development was accomplished by using an enhanced chemiluminescent reagent (EMD Millipore), and band densitometry was achieved with the use of a digitized gel documentation system and associated densitometry software (UVP, Upland, CA, USA). All protein band densities were normalized to Ponceau stain densities. All Western blot analysis data are presented in terms of fold-change from SC rats. Notably, Ponceau staining was used for normalization due to the unknown effects that long-term KD or KS supplementation exerted on putative housekeeping proteins involved in metabolic processes (e.g., GAPDH). Notwithstanding, we have allocated this methodology in past studies examining the effects of KD feeding on skeletal muscle and adipose tissue physiology [9,21,24], and several commentaries exist suggesting that Ponceau normalization for Western blot data is appropriate for models which may affect putative housekeeping protein expression levels [25,26,27].

### 2.8. Tissue Mitochondrial Glutathione Assays in 8 Month-Fed Rat Tissues

Muscle/brain/liver mitochondrial oxidized (GSSG) and total glutathione levels were determined using a commercial colorimetric assay (kit #700190; Cayman Chemical) according to the manufacturer’s instructions, and reduced glutathione (GSH) levels was extrapolated from these values.

### 2.9. Mitochondrial Isolation, Respiration Assays, and Mitochondrial ROS Determination in 8 Month-Fed Rat Tissues

The day of necropsies, differential centrifugation was used to isolate gastrocnemius, brain, and liver mitochondria from fresh tissue using techniques described previously [28,29]. Mitochondrial oxygen consumption was measured as described by Messer et al. [30] in a respiration chamber maintained at 37 °C (Hansatech Instruments, Pentney, King’s Lynn, UK). Isolated mitochondria were incubated with 1 mL of respiration buffer containing (in mM) 100 KCl, 5 KH_2_PO_4_, 1 EGTA, 50 MOPS, 10 MgCl_2_, and 0.2% BSA at 37 °C in a water-jacketed respiratory chamber with continuous stirring. Flux through complex I was measured using 2 mM pyruvate and 2 mM malate, whereas flux through complex II was measured using 5 mM succinate. Rotenone (5 μM) was added to prevent electron backflow to complex I in the succinate-driven experiments. The maximal respiration (state 3), defined as the rate of respiration in the presence of ADP, was initiated by adding 0.25 mM ADP to the respiration chamber containing mitochondria and respiratory substrates. State 4 respiration was recorded following the phosphorylation of ADP. The respiratory control ratio (RCR) was calculated by dividing state 3 by state 4 respiration. 

Mitochondrial ROS production was determined using Amplex red (Molecular Probes, Eugene, OR, USA). The assay was performed at 37 °C in 96-well plates with succinate as the substrate. Specifically, this assay was developed on the concept that horseradish peroxidase catalyzes the H_2_O_2_-dependent oxidation of nonfluorescent Amplex red to fluorescent resorufin red, and it is used to measure H_2_O_2_ as an indicator of superoxide production. SOD was added at 40 U/mL to convert all superoxide to H_2_O_2_. Using a multiwell-plate reader fluorometer (BioTek Synergy H1 Multi-Mode Reader; BioTek), we monitored resorufin formation at an excitation wavelength of 545 nm and a production wavelength of 590 nm. The level of resorufin formation was recorded every 5 min for 15 min, and H_2_O_2_ production was calculated with a standard curve.

### 2.10. Citrate Synthase Activity Assays in 8 Month-Fed Rat Tissues

Muscle/brain/liver tissue homogenate citrate synthase activities were performed as previously described by our laboratory [9]. Briefly, 40 µg of tissue lysate (obtained from tissue lysis described in Section 2.7) were loaded onto 96-well plates. Subsequently, citrate synthase activity was measured as a function of the increase in absorbance from 5,5′-dithiobis-2-nitrobenzoic acid reduction following the methods described elsewhere [31].

### 2.11. Statistical Analysis

All statistical analyses were performed using IBM SPSS version 22.0 (IBM, Armonk, North Castle, NY, USA) and all data are presented as means ± standard error. Statistics on most dependent variables in both experiments were performed using one-way analysis of variance (ANOVA) tests with Tukey post hoc tests being performed when ANOVA *p* < 0.05. A two-way repeated measures ANOVA was performed for rotarod performance data in 8 month-fed rats. If group *p* < 0.05 then a Tukey post hoc test was performed to determine main group effects. If a group × time interaction *p* < 0.05 then: (a) one-way ANOVAs were performed with Tukey post hoc tests in order to determine between-group differences at each time point; and (b) repeated measures ANOVAs were performed within each group and Bonferroni post hoc tests were performed if *p* < 0.05 in order to determine within-group changes over time.

## 3. Results

### 3.1. Effects of Short-Term Feedings on Body Mass Change, Feed Efficiency and Serum BHB Levels

**Figure 1:**
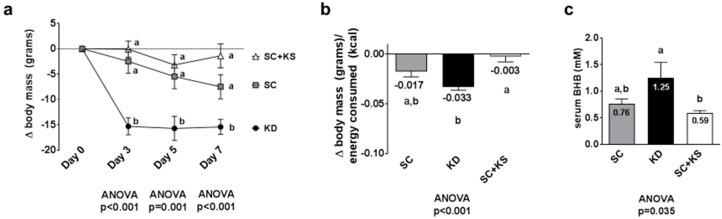
Change in body mass, overall feed efficiency and serum beta-hydroxybutyrate (BHB) levels in 1 week-fed rats. Legend: Change in body mass is presented in panel (**a**). Feed efficiency is presented in panel (**b**). Serum BHB levels are presented in panel (**c**). Data in panel (**a**) are presented as mean ± standard error values (*n* = 10 per group). In panels (**b**,**c**), all bars are presented as mean ± standard error values and group means are indicated within each bar. For all panels, ANOVA *p*-values are presented below data, and different lettering (“a” versus “b”) indicates significant between-group differences whereas shared lettering (“a” as well as “b” or “a, b”) indicates lack of significant between-group differences. Abbreviations: SC, standard chow-fed rats; KD, ketogenic diet-fed rats; SC + KS, standard chow-fed rats with supplemental sodium beta-hydroxybutyrate.

Days 3, 5 and 7 body masses were significantly different between treatments (all ANOVA *p* ≤ 0.001). Specifically, change in body mass at day 3 was significantly lower in KD versus SC and SC + KS rats (*p* < 0.001 and *p* < 0.001, respectively), and this difference persisted at day 5 (*p* = 0.010 and *p* = 0.002, respectively) and day 7 (*p* = 0.036 and *p* < 0.001, respectively) (Figure 1a). Feed efficiency (g body mass gained/kcal consumed) over the 7 days feeding period was significantly different between groups (ANOVA *p* = 0.001). Specifically, KD rats presented significantly lower values compared to SC + KS rats (*p* < 0.001) and differences between KD and SC rats approached significance (*p* = 0.070) (Figure 1b). Serum BHB levels were significantly different between groups (ANOVA *p* = 0.035). Specifically, serum BHB levels were higher in KD versus SC + KS rats (*p* = 0.034), but not different between KD and SC rats (*p* = 0.140) (Figure 1c). Notably, SC + KS rats consumed an average of 0.85 ± 0.03 g/day of sodium beta-hydroxybutyrate over the 1-week intervention through their drinking water.

### 3.2. Effects of Short-Term Feedings on Muscle/Brain/Liver Oxidative Stress-Related mRNAs

**Figure 2:**
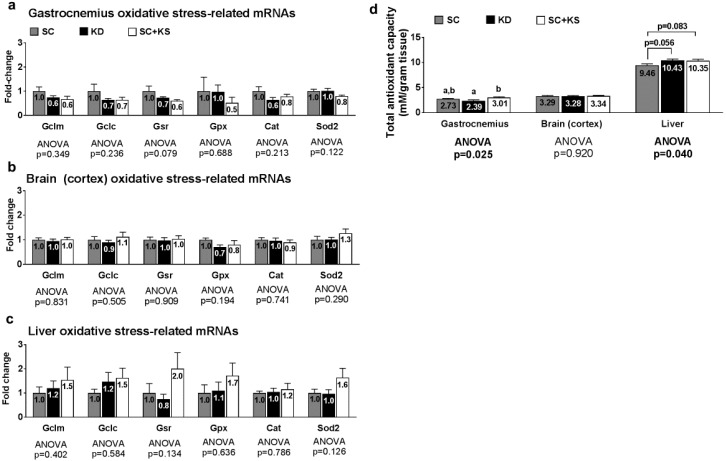
Oxidative stress and endogenous antioxidant-related markers in 1 week-fed rats. Legend: Oxidative stress and endogenous antioxidant related mRNAs are shown for gastrocnemius panel (**a**); brain panel (**b**); and liver panel (**c**). Tissue total antioxidant capacity levels are presented in panel (**d**). Fold-change for each mRNA are expressed relative to the SC group, all bars are presented as mean ± standard error values (*n* = 10 per group), group means are indicated within each bar, ANOVA *p*-values are presented below data, and different lettering in panel D (“a” versus “b”) indicates significant between-group differences whereas shared lettering (“a” as well as “b” or “a, b”) indicates lack of significant between-group differences. Abbreviations: Gclm, glutamate-cysteine ligase modulatory subunit; Gclc, glutamate-cysteine ligase catalytic subunit; Gsr, glutathione reductase; Gpx1, glutathione peroxidase; Cat, catalase; Sod2, supoeroxide dismutase 2; SC, standard chow-fed rats; KD, ketogenic diet-fed rats; SC + KS, standard chow-fed rats with supplemental sodium beta-hydroxybutyrate.

Oxidative stress-related genes (i.e., Gclm, Gclc, Gsr, Gpx1, Cat or Sod2) were not differentially expressed in the gastrocnemius, brain or liver (all ANOVA *p* > 0.05; Figure 2a–c). Gastrocnemius antioxidant capacity was significantly different between groups (ANOVA *p* = 0.025; Figure 2d). Specifically, gastrocnemius antioxidant capacity was significantly lower in KD versus SC + KS rats (*p* = 0.019) but was not different between the KD and SC groups (*p* = 0.275) or SC and SC + KS groups (*p* = 0.366). Brain antioxidant capacity was not different between treatments (ANOVA *p* = 0.920; Figure 2d). Liver antioxidant capacity was significantly different between groups (ANOVA *p* = 0.040), although lower values in the SC group approached but were not significant compared to KD (*p* = 0.056) and SC + KS rats (*p* = 0.083) (Figure 2d).

### 3.3. Effects of Long-Term Feedings on Body Masses, Feed Efficiency and Serum BHB

**Figure 3:**
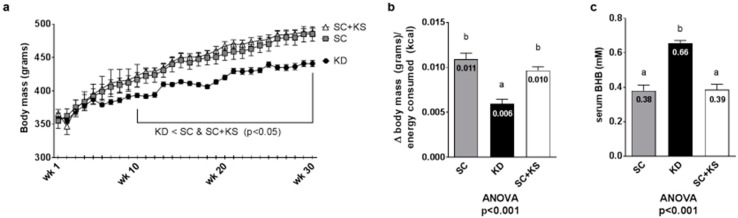
Change in body mass, overall feed efficiency and serum BHB in 8 month-fed rats. Legend: Body mass changes over the intervention are presented in panel (**a**), feed efficiency is presented in panel (**b**) and serum BHB levels are presented in panel (**c**). Data in panel (**a**) are presented as mean ± standard error values (*n* = 7–8 per group). In panels (**b**,**c**), all bars are presented as mean ± standard error values and group means are indicated within each bar. In panels B and C ANOVA *p*-values are presented below data, and different lettering (“a” versus “b”) indicates significant between-group differences whereas shared lettering (“a” as well as “b” or “a, b”) indicates lack of significant between-group differences. Abbreviations: SC, standard chow-fed rats; KD, ketogenic diet-fed rats; SC + KS, standard chow-fed rats with supplemental sodium beta-hydroxybutyrate.

Regarding weekly body masses, there were significant differences between groups from weeks 10–30 (all ANOVA *p* < 0.05). Specifically, KD rats weighed less than SC and SC + KS rats from 10 weeks to 30 weeks (*p* < 0.05 at all time points; Figure 3a). Feed efficiency over the 8 months intervention (i.e., g body mass gained/kcal consumed) was significantly different between groups (ANOVA *p* < 0.001). Specifically, KD rats presented significantly feed efficiency values compared to SC + KS rats (*p* < 0.001) and SC rats (*p* < 0.001) (Figure 3b). Serum BHB levels were significantly different between groups (ANOVA *p* < 0.001). Specifically, serum BHB values were higher in KD versus SC rats (*p* < 0.001) and SC + KS rats (*p* < 0.001) (Figure 3c). Notably, SC + KS rats consumed an average of 0.21 ± 0.02 g/day of sodium beta-hydroxybutyrate salts through their drinking water over the 8 months intervention.

### 3.4. Effects of Long-Term Feedings on Rotarod Performance

**Figure 4:**
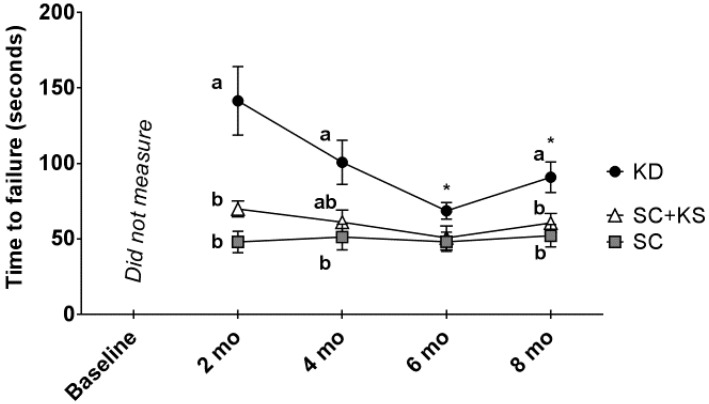
Change in rotarod performance in 8 month-fed rats. Legend: Rotarod performance every 2 months are presented herein. All data are presented as mean ± standard error values (*n* = 7–8 per group). Given the significant group × tine interaction, post hoc analyses at each time point and within each group were performed. Specifically, different lettering (“a” versus “b”) indicates significant between-group differences whereas shared lettering (“a” as well as “b” or “a, b”) indicates lack of significant between-group differences, and asterisks indicate significant decreases within the KD rats at 6 and 8 months compared to 2 months values (*p* < 0.05). Abbreviations: SC, standard chow-fed rats; KD, ketogenic diet-fed rats; SC + KS, standard chow-fed rats with supplemental sodium beta-hydroxybutyrate.

There was a significant main group effect (*p* < 0.001) and group × time interaction (*p* = 0.005) for rotarod performance (Figure 4). Regarding the main group effect, rotarod performance was significantly higher in KD rats versus SC (*p* = 0.001) and SC + KS rats (*p* = 0.005). Regarding the group × time interaction: (a) at 2 months, performance was significantly higher in the KD versus the SC (*p* < 0.001) and SC + KS groups (*p* = 0.009); (b) at 4 months, performance was significantly higher in the KD versus the SC (*p* = 0.017) group, and values between the KD versus SC + KS group approached significance (*p* = 0.060); (c) at 8 months, performance was significantly higher in the KD versus the SC (*p* = 0.008) and SC + KS groups (*p* = 0.049); and (d) within the KD group there was a significant time effect (*p* = 0.001) whereby values at 6 months (*p* = 0.006) and 8 months (*p* = 0.021) were significantly lower than 2 months values (notably, there was no time effect within the SC (*p* = 0.531) and SC + KS groups (*p* = 0.382).

### 3.5. Effects of Long-Term Feedings on Gastrocnemius Oxidative Stress-Related Proteins and Markers

**Figure 5:**
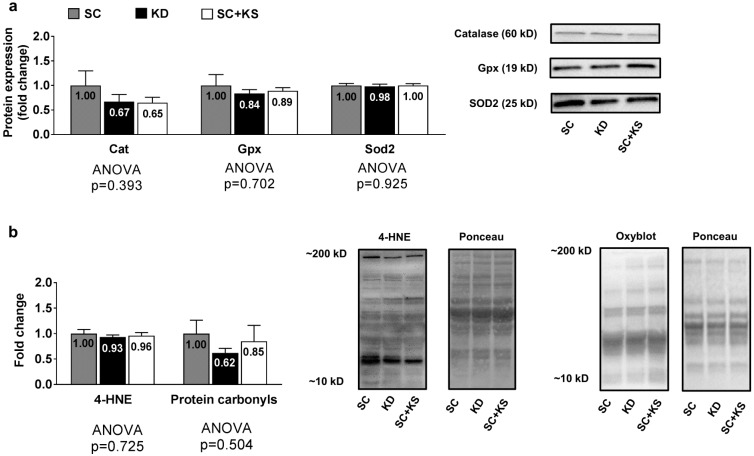
Gastrocnemius oxidative stress-related proteins and markers in 8 month-fed rats. Legend: Gastrocnemius catalase (Cat), glutathione peroxidase (Gpx), and mitochondrial superoxide dismutase (Sod2) protein levels are presented with representative images in panel (**a**). In panel (**b**), tissue 4-hydroxynonenal (4-HNE) and protein carbonyls (Oxyblot) are presented with representative images. Fold-change for each protein are expressed relative to the SC group, all bars are presented as mean ± standard error values (*n* = 7–8 per group), group means are indicated within each bar and ANOVA *p*-values are presented below data. Abbreviations: SC, standard chow-fed rats; KD, ketogenic diet-fed rats; SC + KS, standard chow-fed rats with supplemental sodium beta-hydroxybutyrate.

Gastrocnemius protein expression levels of Cat (ANOVA *p* = 0.393), Gpx (ANOVA *p* = 0.702) and Sod2 (ANOVA *p* = 0.925) were not differentially expressed between groups (Figure 5a). Likewise, gastrocnemius 4-HNE (ANOVA *p* = 0.725) and protein carbonyl levels (ANOVA *p* = 0.504) were not differentially expressed groups (Figure 5b). 

### 3.6. Effects of Long-Term Feedings on Brain Oxidative Stress-Related Proteins and Markers

**Figure 6:**
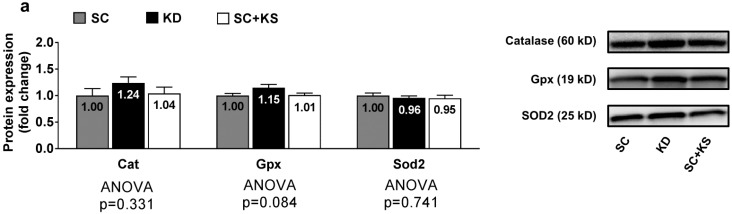

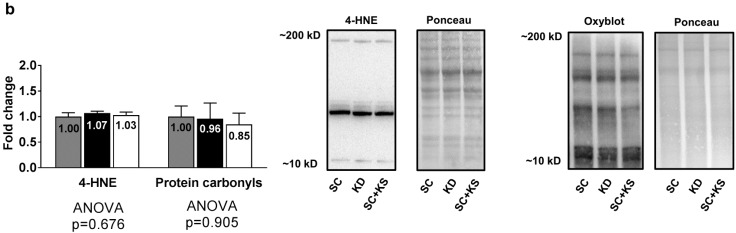
Brain oxidative stress-related proteins and markers in 8 month-fed rats. Legend: Brain catalase (Cat), glutathione peroxidase (Gpx), and mitochondrial superoxide dismutase (Sod2) protein levels are presented with representative images in panel (**a**). In panel (**b**), tissue 4-Hydroxynonenal (4-HNE), and protein carbonyls (Oxyblot) are presented with representative images. Fold-change for each protein are expressed relative to the SC group, all bars are presented as mean ± standard error values (*n* = 7–8 per group), group means are indicated within each bar and ANOVA *p*-values are presented below data. Abbreviations: SC, standard chow-fed rats; KD, ketogenic diet-fed rats; SC + KS, standard chow-fed rats with supplemental sodium beta-hydroxybutyrate.

Brain protein expression levels of Cat (ANOVA *p* = 0.331), Gpx (ANOVA *p* = 0.084) and Sod2 (ANOVA *p* = 0.741) were not differentially expressed between groups (Figure 6a). Likewise, brain 4-HNE (ANOVA *p* = 0.676) and protein carbonyl levels (ANOVA *p* = 0.905) were not differentially expressed between groups (Figure 6b). 

### 3.7. Effects of Long-Term Feedings on Liver Oxidative Stress-Related Proteins and Markers

**Figure 7:**
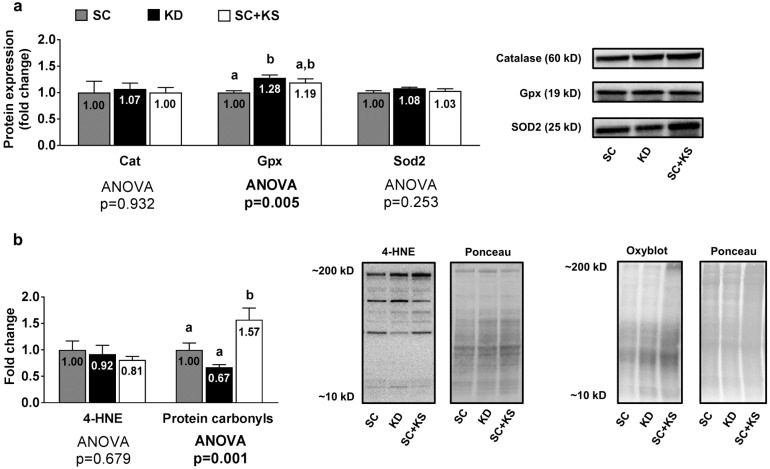
Liver oxidative stress-related proteins and markers in 8 month-fed rats. Legend: Liver catalase (Cat), glutathione peroxidase (Gpx), and mitochondrial superoxide dismutase (Sod2) protein levels are presented with representative images in panel (**a**). In panel (**b**), tissue 4-Hydroxynonenal (4-HNE), and protein carbonyls (Oxyblot) are presented with representative images. Fold-change for each protein are expressed relative to the SC group, all bars are presented as mean ± standard error values (*n* = 7–8 per group), group means are indicated within each bar, ANOVA *p*-values are presented below data and different lettering (“a” versus “b”) indicates significant between-group differences whereas shared lettering (“a” as well as “b” or “a, b”) indicates lack of significant between-group differences. Abbreviations: SC, standard chow-fed rats; KD, ketogenic diet-fed rats; SC + KS, standard chow-fed rats with supplemental sodium beta-hydroxybutyrate.

Liver protein expression levels of Gpx were greater in the KD versus the SC group (*p* = 0.004) and high levels approached significance in the KD versus the SC + KS group (*p* = 0.068) (Figure 7a). Liver protein expression levels of Cat (ANOVA *p* = 0.932) and Sod2 (ANOVA *p* = 0.253) were not differentially expressed between groups (Figure 7a). Liver 4-HNE levels were not differentially expressed between groups (ANOVA *p* = 0.684), although liver protein carbonyls were greater in the SC + KS versus SC (*p* = 0.034) and KD (*p* = 0.001) groups (Figure 7b). 

### 3.8. Effects of Long-Term Feedings on Tissue Mitochondrial Glutathione, Total Antioxidant Capacity, and ROS Levels

**Figure 8:**
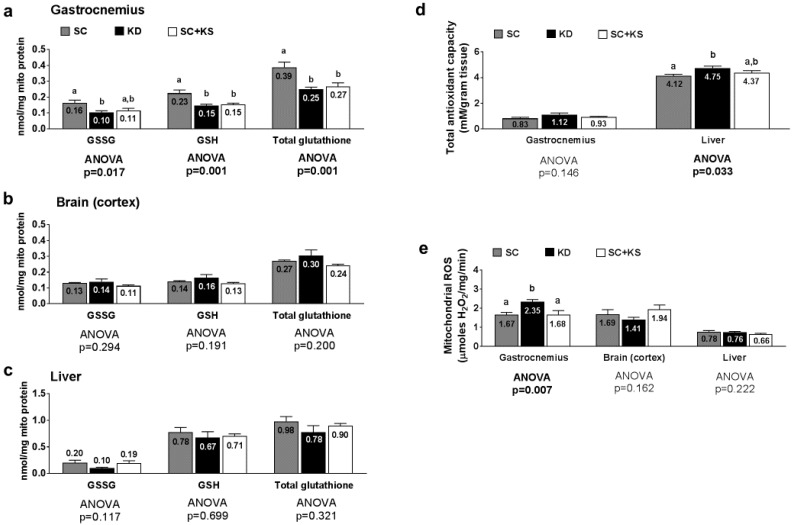
Tissue mitochondrial glutathione, total antioxidant capacity, and ROS levels in 8 month-fed rats. Legend: Gastrocnemius mitochondrial oxidized (GSSG), reduced (GSH) and total glutathione are presented in panel (**a**); Brain mitochondrial oxidized (GSSG), reduced (GSH) and total glutathione are presented in panel (**b**); Liver mitochondrial oxidized (GSSG), reduced (GSH) and total glutathione are presented in panel (**c**); Tissue total antioxidant capacity levels are presented in panel (**d**); Tissue mitochondrial reactive oxygen species production levels are presented in panel (**e**). All bars are presented as mean ± standard error values (*n* = 7–8 per group), group means are indicated within each bar and ANOVA *p*-values are presented below data, and different lettering (“a” versus “b”) indicates significant between-group differences whereas shared lettering (“a” as well as “b” or “a, b”) indicates lack of significant between-group differences. Abbreviations: SC, standard chow-fed rats; KD, ketogenic diet-fed rats; SC + KS, standard chow-fed rats with supplemental sodium beta-hydroxybutyrate.

Gastrocnemius mitochondrial oxidized (GSSG) glutathione, reduced (GSH) glutathione and total glutathione were differentially expressed between groups (ANOVA *p* values = 0.001–0.017; Figure 8a). Specifically, the following was found to occur: (a) GSSG levels were greater in SC-fed versus KD-fed rats (*p* = 0.018) and trended higher in SC-fed versus SC + KS-fed rats (*p* = 0.063); (b) GSH levels were greater in the SC versus the KD (*p* = 0.001) and SC + KS (*p* = 0.004) groups; and (c) total glutathione levels were greater in the SC versus the KD (*p* = 0.001) and SC + KS (*p* = 0.005) groups. Brain mitochondrial GSSG (ANOVA *p* = 0.294), GSH (ANOVA *p* = 0.191) and total glutathione (ANOVA *p* = 0.200) were not differentially expressed between groups (Figure 8b). Liver mitochondrial GSSG (ANOVA *p* = 0.117), GSH (ANOVA *p* = 0.699) and total glutathione (ANOVA *p* = 0.321) were not differentially expressed between groups (Figure 8c). Gastrocnemius total antioxidant capacity levels were not different between groups (ANOVA *p* = 0.146; Figure 8d). Liver total antioxidant capacity levels were significantly higher in KD versus SC rats (*p* = 0.028), but not KD versus SC + KS rats (*p* = 0.216; Figure 8d). Gastrocnemius mitochondrial ROS production was significantly different between groups (ANOVA *p* = 0.007; Figure 8e). Specifically, ROS values were significantly higher in KD rats versus the SC (*p* = 0.014) and SC + KS groups (*p* = 0.015). Mitochondrial ROS production in the brain (ANOVA *p* = 0.162) and liver (ANOVA *p* = 0.222) were not different between groups (Figure 8e).

### 3.9. Effects of Long-Term Feedings on Gastrocnemius Mitochondrial Function

**Figure 9:**
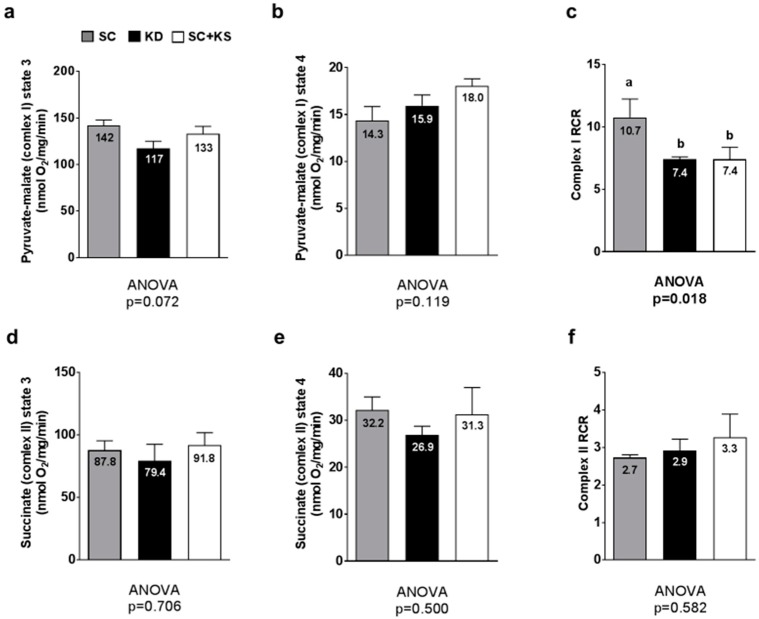
Gastrocnemius mitochondrial function in 8 month-fed rats. Legend: State 3 pyruvate-malate is presented in panel (**a**); State 4 pyruvate-malate is presented in panel (**b**); Pyruvate-malate RCR is presented in panel (**c**); State 3 succinate is presented in panel (**d**); State 4 succinate is presented in panel (**e**); Succinate RCR is presented in panel (**f**). All bars are presented as mean ± standard error values (*n* = 7–8 per group), group means are indicated within each bar and ANOVA *p*-values are presented below data, and different lettering (“a” versus “b”) indicates significant between-group differences whereas shared lettering (“a” as well as “b” or “a, b”) indicates lack of significant between-group differences. Abbreviations: SC, standard chow-fed rats; KD, ketogenic diet-fed rats; SC + KS, standard chow-fed rats with supplemental sodium beta-hydroxybutyrate; RCR, respiratory exchange ratio.

Between-group differences in gastrocnemius pyruvate-malate state 3 approached significance (ANOVA *p* = 0.072) whereby lower values in KD versus SC rats approached significance (*p* = 0.064) (Figure 9a). Gastrocnemius pyruvate-malate state 4 was not different between groups (*p* = 0.119; Figure 9b). Gastrocnemius pyruvate-malate RCR values were significantly different between groups (ANOVA *p* = 0.018). Specifically, values were significantly lower in KD versus SC rats (*p* = 0.030) and SC + KS versus SC rats (*p* = 0.035) (Figure 9c). Gastrocnemius succinate state 3 (ANOVA *p* = 0.706; Figure 9d), succinate state 4 (ANOVA *p* = 0.500; Figure 9e) and succinate RCR values (ANOVA *p* = 0.582; Figure 9f) were not different between groups.

### 3.10. Effects of Long-Term Feedings on Brain and Liver Mitochondrial Function

**Figure 10:**
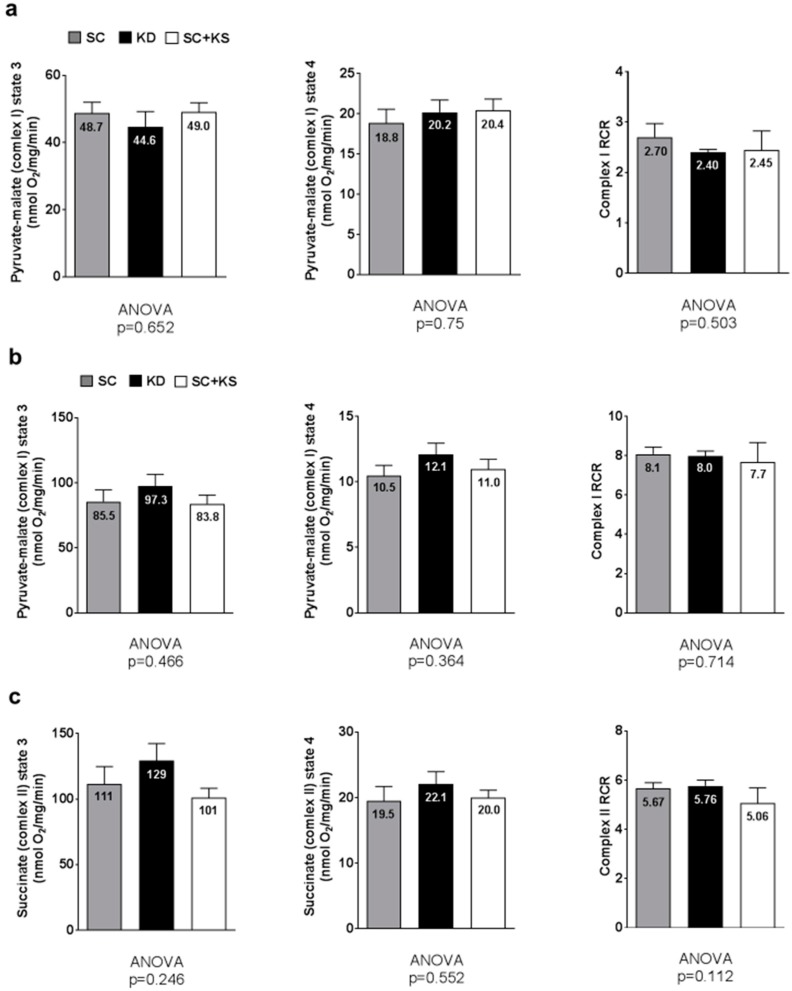
Brain and liver mitochondrial function in 8 month-fed rats. Legend: Brain state 3 and 4 pyruvate-malate and respiratory control ratio (RCR) values are presented in panel (**a**); Liver state 3 and 4 pyruvate-malate and RCR values are presented in panel (**b**); Liver state 3 and 4 succinate and RCR values are presented in panel (**c**). All bars are presented as mean ± standard error values (*n* = 7–8 per group), group means are indicated within each bar and ANOVA *p*-values are presented below data. Abbreviations: SC, standard chow-fed rats; KD, ketogenic diet-fed rats; SC + KS, standard chow-fed rats with supplemental sodium beta-hydroxybutyrate; RCR, respiratory exchange ratio.

Brain pyruvate-malate state 3 (ANOVA *p* = 0.706), pyruvate-malate state 4 (ANOVA *p* = 0.500) and pyruvate-malate RCR values (ANOVA *p* = 0.582; Figure 10a) were not different between groups. Notably, brain succinate state 3, state 4 and RCR values are not reported given that a reliable state 4 could not be obtained. 

Liver pyruvate-malate state 3 (ANOVA *p* = 0.466), pyruvate-malate state 4 (ANOVA *p* = 0.364) and pyruvate-malate RCR values (ANOVA *p* = 0.714; Figure 10b) were not different between groups. Liver succinate state 3 (ANOVA *p* = 0.246), succinate state 4 (ANOVA *p* = 0.552) and succinate RCR values (ANOVA *p* = 0.112; Figure 10c) were not different between groups.

### 3.11. Effects of Long-Term Feedings on Tissue Citrate Synthase Activity

**Figure 11:**
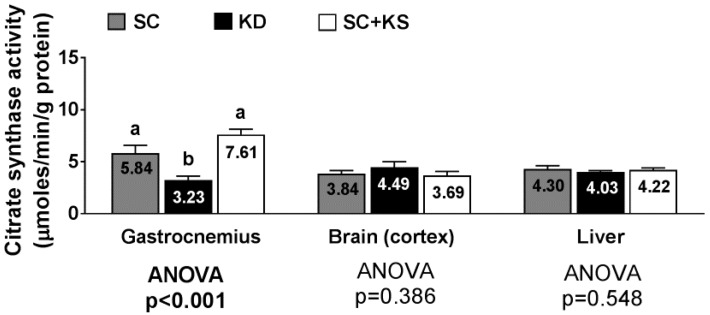
Tissue citrate synthase activity in 8 month-fed rats. Legend: Tissue citrate synthase activity is presented. All bars are presented as mean ± standard error values (*n* = 7–8 per group), group means are indicated within each bar and ANOVA *p*-values are presented below data, and different lettering (“a” versus “b”) indicates significant between-group differences whereas shared lettering (“a” as well as “b” or “a, b”) indicates lack of significant between-group differences. Abbreviations: SC, standard control; KD, ketogenic diet; SC + KS, standard control with supplemental sodium beta-hydroxybutyrate.

Gastrocnemius citrate synthase activity levels were significantly different between groups (ANOVA *p* < 0.001). Specifically, values were lower in KD versus SC + KS (*p* < 0.001) and SC (*p* = 0.009) rats (Figure 11). Citrate synthase activity levels in the brain (ANOVA *p* = 0.386) and liver (ANOVA *p* = 0.548) were similar between groups (Figure 11).

## 4. Discussion

Overall, KD-fed rats exhibited more weight loss (1 week feedings) or an attenuation of weight gain (8 months feedings) compared to the SC and SC + KS groups. In short-term fed rats, serum ketones and liver antioxidant capacity trended higher in KD- versus SC-fed rats, although all muscle, brain and liver oxidative stress-related mRNAs were similar between treatments. In long term-fed rats, serum ketones were greater in KD- versus SC- and SC + KS-fed rats (*p* < 0.05) suggesting that diet, but not the employed KS dosage, induces ketonemia. Moreover, liver total antioxidant capacity and glutathione peroxidase protein levels were 15% and 28% higher, respectively, in long-term KD- versus SC-fed rats (*p* < 0.05), while liver protein carbonyls were lowest in KD-fed rats. Contrary to our hypothesis, gastrocnemius mitochondrial ROS production was ~40% higher in KD-fed rats versus other groups (*p* = 0.007) and this may have been related to significantly lower mitochondrial glutathione levels. Further, pyruvate-malate mitochondrial respiratory control ratio was lower in KD- and SC + KS- versus SC-fed rats (*p* < 0.05), and gastrocnemius mitochondrial quantity was 81% and 136% greater in SC- and SC + KS-fed rats versus KD-fed rats (*p* < 0.001). Rotarod performance was greatest in KD-fed rats versus other groups at 2, 4 and 8 months (*p* < 0.05), although an age-related decline in performance existed in KD-fed rats whereas this was not evident in the other two groups.

### 4.1. Ketogenic Diet Feeding, But Not Ketone Salt Supplementation, Elevates Serum BHB Levels and Produces Short-Term Weight Loss as Well as a Long-Term Attenuation of Weight Gain

Herein, we report that KD feeding induces short-term weight loss and reduced feed efficiency relative to standard chow feeding. Notably, SC-fed rats also presented weight loss during the 1-week feeding experiment, although KD-induced weight loss was significantly greater. While this finding is difficult to reconcile, we believe that the initial handling of rats (e.g., body mass assessment every other day and food weighing every day) was likely a stressor to all groups of rats in the 1-week study. Hence, we are uncertain as to whether or not this stressor appreciably compromised the molecular markers examined in the 1-week animals. In spite of the aforementioned limitation, our group has previously observed similar outcomes in a separate cohort of rats fed the unmodified KD (i.e., no additional protein or fiber added) over a 6-week period when compared to SC-fed and Western diet-fed rats [24]. Likewise, other studies have reported similar findings using ketogenic diets in rodents [32,33,34]. While the particular biochemical cascade and subsequent hormonal milieu that occurred during this timeframe was beyond the scope of this study, KD-induced weight loss (or weight gain prevention) may be prompted by chronically low insulin levels and/or due to the bodily loss of biochemical energy via ketone body excretion in the urine [35]. Notably, ketone salt supplementation did not affect body weight or feed efficiency when comparing SC + KS versus SC-fed rats. Ketone ester feedings in mice over a 4-week period has been shown to robustly elevate serum BHB levels, stimulate brown fat activation, elevate resting energy expenditure and promote weight loss in mice [36]. Kesl et al. [37] also reported that 5 and 10 g/kg of KS, which is 8-to-16-fold greater than our employed KS dosage, prevents weight gain in rats although these dosages did not alter serum BHB levels. Providing a mechanistic explanation as to why serum BHB levels are not responsive to exogenous KS versus ketone ester supplementation in rats is difficult to reconcile, but we posit that the lack of significant physiological effects of KS supplementation in both feeding experiments are likely due to the relatively lower KS doses employed in the current study.

### 4.2. Short-Term Ketogenic Diet Feeding or Ketone Salt Supplementation Do Not Alter Oxidative Stress-Related Gene Expression in Muscle/Brain/Liver Tissue

The purpose of the 1 week experiment was to test the theory suggested by Milder and Patel [8] whereby ketogenic diet feeding is posited to increase the mRNA expression of glutathione-related mRNAs (i.e., Gclc and Gclm) after a one week period leading to an enhanced endogenous antioxidant defense system. Additionally, we were interested in examining the effects of KS supplementation given recent findings like that of Shimazu et al. [38] who have reported that BHB acts as an antioxidant in vitro. The data presented herein do not demonstrate altered mRNA expression of either Gclc or Gclm in skeletal muscle, whole brain or liver tissue after 1 week of KD or KS feeding in rats. In addition, we did not observe mRNA alterations in other endogenous antioxidants (i.e., Gsr, Gpx1 and Sod2) or total antioxidant capacity in the assayed tissues. In contrast to our findings, Jarrett and colleagues [14] observed increased GCL activity and elevated protein levels in response to a 3 weeks of KD feeding in rat hippocampal tissue. Discrepancies in our outcomes related to KD feeding could be due to: (a) our investigation of mRNA markers following a one week time course; and/or (b) with regard to our findings in the brain, discordant findings may be related to the region of the brain that was assayed. Notably, we performed all assays in the whole cortical brain while others examining KD-induced alterations in mRNA and protein/enzyme activity have examined the hippocampus. For instance, Ziegler and colleagues [15] also reported that KD feeding over an 8 week period in rats increased Gpx activity increased in the hippocampus, reduced Cat activity in the cerebellum and did not alter these markers in the cortex. Thus, these data collectively suggest that: (a) different brain regions likely express different biochemical responses to KD feeding; and (b) short- or long-term KD feeding does not appreciably alter oxidative stress-related gene expression in skeletal muscle or liver tissue. The lack of appreciable antioxidant effects regarding short-term KS supplementation is likely due to the employed dosage not eliciting increases in serum BHB levels as stated above. 

### 4.3. Long-Term Ketogenic Diet Feeding Positively Impacts Select Markers of Oxidative Stress in the Liver But Does Not Alter Mitochondrial Quality in Liver or Brain Tissue

Our findings that the short- and long-term KD feeding increases liver total antioxidant capacity, and long-term KD feeding increases Gpx protein expression levels and decreases liver protein carbonyl levels (numerically versus SC-fed rats and significantly versus SC + KS-fed rats) indicate a potential protective effect of the KD feeding on liver tissue. Several studies have observed that KD feeding increase inflammation and fibroblast growth factor 21-mediated hepatocyte steatosis in rodents [3,39,40]. However, we have previously reported decreased liver inflammatory signaling and triglyceride accumulation with KD feeding in rats whereby the KD was similar to the one utilized in the present study [24]. Milder et al. [18] have also reported an increase in nuclear Nrf2 and a decrease in liver mitochondrial glutathione levels following 3 weeks of KD feeding in rats. While we did not assess nuclear Nrf2 levels in the current study and report that the long-term KD feeding does not alter liver glutathione levels, the contention by Milder et al. that short-term KD feeding leads to long-term increases in oxidative stress defenses may be a potential explanation as to why protein carbonyls were lowest in long-term KD fed animals while Gpx protein and total antioxidant levels were relatively higher in these same animals. It is also plausible that lower liver inflammatory signaling and/or lower triglyceride accumulation, as reported in our past study, could be a contributing mechanism to increased liver oxidative stress protection given the link between ectopic tissue fat accumulation, inflammation and oxidative stress [41]. 

Interestingly, we also observed that long-term ketone salt supplementation significantly increased liver protein carbonyl levels. While there is currently no literature to date documenting such effects, this suggests that the daily low-dose consumption of ketone salts may induce repetitive bouts of oxidative stress to the liver and this finding warrants further mechanistic investigation. It should also be noted that unaltered brain and liver citrate synthase activity (a well-validated surrogate of mitochondrial density [42]) in KD-fed rats is also discordant with previous literature given that increases in citrate synthase activity have been reported to occur in the hippocampus of KD-fed rodents after a 3-week period [10,43]. Again, however, discrepancies in our findings versus the aforementioned literature may be explained by the long-term nature of our study as well as tissue-specific differences that occur in response to ketogenic diet feedings. Beneficial KD-induced mitochondrial adaptations that occur in the liver or brain, if they do occur, may be transient once the rodents are given an extended period time for dietary adaptation.

### 4.4. Long-Term Ketogenic Diet Feeding Negatively Impacts Skeletal Muscle Mitochondrial Physiology

While long-term KD feeding did not alter markers of brain and liver mitochondrial quality or quantity, we observed that long-term KD feeding robustly impacts several of these variables in skeletal muscle. Specifically, long-term KD-fed rats expressed lower gastrocnemius glutathione levels, lower gastrocnemius maximal citrate synthase activity, higher gastrocnemius mitochondrial ROS production, and an impairment in gastrocnemius pyruvate-malate respiratory control. Our laboratory has previously investigated skeletal muscle mitochondrial density and mitochondrial quality in 3 months old KD-fed rats following a 6 week dietary intervention [9]. We reported that succinate respiratory control increased in KD-fed rats compared to rats fed a Western diet, suggesting that shorter-term ketogenic dieting may improve lipid oxidation. However, the present study indicates that this mitochondrial adaptation does not persist after 8 months of KD feeding. Regarding the pyruvate-malate respiratory control ratio in skeletal muscle, the depression observed in KD-fed rats may be related to increased concentrations of uncoupling proteins in the gastrocnemius mitochondria. This phenomenon has been observed by others who reported KD-induced uncoupling protein expression increases in brown adipose and neural tissue, respectively [13,44]. Likewise, the KD-induced decrease in gastrocnemius mitochondrial pyruvate-malate respiratory control ratio may also be related to the increased ROS production observed given that ROS-induced mitochondrial damage is implicated in the reduction of mitochondrial function [45,46,47]. The KD-induced decrease in gastrocnemius citrate synthase activity observed may also be indicative of decrements in skeletal muscle mitochondrial quality or quantity. In this regard, past literature suggests that ketonemia, high fat diet feeding and/or fasting in humans and rodents down-regulates oxidative phosphorylation, lowers skeletal muscle mitochondrial density, or lowers the efficiency mitochondrial respiration [48,49,50]. The KD-induced increase in gastrocnemius mitochondrial ROS and/or decrease in mitochondrial function and density may have also been due to mitochondrial glutathione levels being low in KD-fed animals. In support of this hypothesis, others have reported that low mitochondrial glutathione levels are associated with increased ROS production and decrements in mitochondrial function [51,52]. Collectively, our results along with other observations in past studies suggest that while short-term ketogenic dieting may enhance mitochondrial biogenesis and/or alter mitochondrial physiology in certain tissues, skeletal muscle may be particularly susceptible to KD-induced alterations in mitochondrial physiology following long-term feeding, and this phenomenon may be due to KD-induced decrements in mitochondrial glutathione levels and/or another unidentified mechanism. 

It is notable that, despite the aforementioned observations in the gastrocnemius, KD-fed rats exhibited higher rotarod performance throughout the long-term intervention relative to the other two groups. While rotarod testing does not test maximal aerobic capacity, it does provide a reliable measure of coordination, muscle strength and endurance [23]. Therefore, our rotarod performance data suggest that long-term ketogenic dieting may result in increased skeletal muscle metabolic efficiency (i.e., similar or improved work output with lowered mitochondrial function and/or density in muscle). However, it is notable that rotarod performance robustly decreased with age in KD-fed rats, whereas this did not occur in the SC and SC + KS groups; this being suggestive that long-term KD-induced decrements in mitochondrial function may be a contributor to age-related declines in rotarod performance. In this regard, further research is needed in order to examine: (a) the KD-associated mechanisms that affect mitochondrial function and/or density; and/or (b) if KD feeding affects metabolic efficiency in skeletal muscle.

### 4.5. Experimental Considerations

Certain limitations are evident in the current study. First, due to resource constraints, short-term tissue analyses herein were only performed at one sacrificial time point which differs from Milder et al. [18] who examined the effects of ketogenic dieting at 1-day, 3-day, 1-week and 3-week sacrificial time points. Second, and as mentioned prior, our brain assays were performed on cortical brain lysates; this is an experimental consideration which likely masked any potential benefit of KD feeding and/or ketone salt supplementation on the hippocampus or other brain regions. Third, it is notable that total antioxidant capacity levels were assayed in different tissues rather than individual antioxidant enzyme activities due to resource constraints. Thus, given that liver antioxidant capacity increases in response to long-term ketogenic dieting, studying the effects of ketogenic dieting on single antioxidant activity levels are warranted. Lastly, it is noteworthy that sedentary rats were studied herein, and this may be a potential reason as to why skeletal muscle mitochondria is negatively impacted with long-term KD feeding. In this regard, we have previously reported that increases in skeletal muscle mitochondrial quality and quantity are not impaired in KD-fed rats that exercised via resistance-loaded voluntary wheel running over a 6-week period [9]. Moreover, elite ultra-endurance athletes that chronically engage in ketogenic-like dieting have been reported to perform equally as well in laboratory-based exercise tests compared to athletes that consume high-carbohydrate diets [53]; this is suggestive that long-term KD athletes do not present functional impairments in skeletal muscle mitochondrial integrity. Hence, again, a future research aim would be to examine if skeletal muscle mitochondrial deficits observed in 8 months KD-fed rats are mitigated with exercise training. One final experimental consideration is the rapid weight loss observed in one long-term SC + KS rat as well as the tumor observed during dissections in one long-term SC-fed rat. While the mechanistic causes of both events were not more thoroughly explored, it is notable that no adverse events were observed in KD-fed rats. Hence, long-term KD feeding in rodents appears to be safe and, with regard to the latter observation, future research is needed in order to determine if KD feeding has anti-tumorigenic effects. 

## 5. Conclusions

In spite of the aforementioned limitations, we posit that these data provide a comprehensive evaluation as to how short-term and long-term KD feeding affect oxidative stress markers, endogenous antioxidant gene and protein expression and mitochondrial function in multiple tissues in rats. Longer-term human studies examining muscle biopsy specimens are needed in order to validate our findings, suggesting that long-term KD feeding appreciably alters mitochondrial physiology.
